# Cilia Distal Domain: Diversity in Evolutionarily Conserved Structures

**DOI:** 10.3390/cells8020160

**Published:** 2019-02-14

**Authors:** Helena Soares, Bruno Carmona, Sofia Nolasco, Luís Viseu Melo, João Gonçalves

**Affiliations:** 1 Center for Chemistry and Biochemistry, Faculty of Sciences, University of Lisbon, 1749-016 Lisbon, Portugal; bfcarmona@fc.ul.pt; 2 Health Technology College of Lisbon (ESTeSL)—Polytechnic Institute of Lisbon, 1990-096 Lisbon, Portugal; sofianolasco@fmv.ulisboa.pt; 3 CIISA-Interdisciplinary Centre of Research in Animal Health, Faculty of Veterinary Medicine, University of Lisbon, 1300-666 Lisbon, Portugal; 4 Physics Department and CEFEMA, IST, University of Lisbon, Av. Rovisco Pais, 1049-001 Lisboa, Portugal; luis.melo@tecnico.ulisboa.pt; 5 Deep Genomics, MaRS Centre, 661 University Ave, Suite 480, Toronto, ON M5G 1M1, Canada; joao.alg@gmail.com

**Keywords:** cilia, cilia distal domain, microtubule-capping structures, cilia structural diversity

## Abstract

Eukaryotic cilia are microtubule-based organelles that protrude from the cell surface to fulfill sensory and motility functions. Their basic structure consists of an axoneme templated by a centriole/basal body. Striking differences in ciliary ultra-structures can be found at the ciliary base, the axoneme and the tip, not only throughout the eukaryotic tree of life, but within a single organism. Defects in cilia biogenesis and function are at the origin of human ciliopathies. This structural/functional diversity and its relationship with the etiology of these diseases is poorly understood. Some of the important events in cilia function occur at their distal domain, including cilia assembly/disassembly, IFT (intraflagellar transport) complexes’ remodeling, and signal detection/transduction. How axonemal microtubules end at this domain varies with distinct cilia types, originating different tip architectures. Additionally, they show a high degree of dynamic behavior and are able to respond to different stimuli. The existence of microtubule-capping structures (caps) in certain types of cilia contributes to this diversity. It has been proposed that caps play a role in axoneme length control and stabilization, but their roles are still poorly understood. Here, we review the current knowledge on cilia structure diversity with a focus on the cilia distal domain and caps and discuss how they affect cilia structure and function.

## 1. Introduction

### The Discovery of the Basic Cilia Structure at a Glance

Eukaryotic cilia/flagella are fascinating microtubule-based organelles that protrude from the cell surface. These organelles, composed of more than 1000 polypeptides [1,2], are complex compartments that captured the attention of cell biologists since they were first observed under a microscope. In fact, van Leeuwenhoek (1676) observing protozoa cells described them as “animalcules … with …little feet, or little legs, which were moved…” [3]. The observation of these intriguing structures, both in protozoa and in spermatozoa, led to the idea that cilia have a permanent fibrillar structure arranged with its long axis parallel to that of the cilium, and that is at the base of cilia movements by contraction [4]. 

The advent of electron microscopy (EM) created new opportunities to study the ciliary structure. EM observations of cilia/flagella from a number of flagellates, algae, fungi and human further showed that cilia were composed of multiple fibrils enclosed in a common matrix of protoplasm and surrounded by a membrane [5,6,7,8,9]. However, only in 1954, comparative studies by Fawcett and Porter [10] of thin sections of mollusk, amphibian, mouse and human cilia showed that these present an internal structure (axoneme) built of a ring of nine double filaments along the periphery and a central pair of single filaments. It also became clear that cilia emanate from complex elongated basal corpuscles/basal bodies from which ciliary rootlets (striated fibers) extend downward into the cytoplasm likely to anchor them [10]. A Similar arrangement of the axonemal filamentous components were observed in the cilia and flagella of plants [9], strongly suggesting that it could be conserved throughout the phylogenetic tree. 

The advances on knowledge about the ciliary structure cannot be separated from structural and functional studies on the centrosome. The idea that centrioles are basically the same organelles as basal bodies was proposed in the second half of the 19th century [11]. This proposal triggered intense debate, and only much later, further EM studies permitted to establish the structural identity of basal bodies and centrioles and the confirmation that they are identical [11]. Later, it was shown that the injection of *Xenopus* eggs with purified basal bodies was able to replace centrioles in aster formation [12]. However, despite the body of work concerning the structure, function and biogenesis of centrosomes and basal bodies developed since, how a centriole becomes a basal body is still a process not fully understood. 

The progressive improvement of preservation/fixation and negative staining techniques for EM samples, allowed detailed observations of the axoneme structures of motile cilia. This led to the description of new axonemal elements such as side arms and bridges, between the peripheral filaments of the axoneme, as well as radial spokes [13,14,15]. The first basic reconstruction of a flagellum structure from the tip to the basal body, along the axoneme, could thus be obtained, and showed the existence of structural diversity between different cilia types and species [16].

Although a detailed ciliary structure was emerging, the biochemical characterization of the ciliary compartment was lagging behind. Parallel to the characterization of cilia, elegant EM studies started to reveal other cellular structures more accurately. For example, microtubules were shown to be associated with centrioles, the mitotic apparatus and the midbody during cytokinesis [17,18,19] in diverse cell types. Their structural resemblance with the protofibrils composing the nine peripheral and central filaments of the axoneme (9+2 structure) of the human and rat sperm tails and *Tetrahymena* cilia [20,21] suggested a microtubule-based nature for the axoneme, and a role for these polymers in cilia movement [22]. Later, efforts to identify ciliary proteins revealed the microtubule building block tubulin and the microtubule motor protein dynein as major components of these organelles [23,24,25,26].

The existence of cilia (primary cilia), distinct from the motile cilia previously observed in protists and multiciliated tissues, were noticed in the 19th century, but it was Zimmermann (1898) that described them in mammalian cells. He designated these cilia as ‘centralgeissel’ (central flagella), showed they were associated with a pair of centrioles (the diplosome), and proposed a sensory role for them [11]. In 1961, Barnes also reports the presence of solitary non-motile cilia in a great variety of somatic cells. She showed that these projected from one of the centrioles of the centrosome and lacked the central pair of axial fibers characteristic of motile cilia. Furthermore, she proposed the existence of a correlation between the (9+2) fibers‘ organization pattern with motile cilia, which are organized from a single centriole or basal body. The presence of (9+0) cilia in sensory cells resurfaced the idea that they could have sensory functions [27]. There was, however, debate on the nature of immotile, or primary cilia. For example, in 1962, Sorokin suggested that the structural arrangement of these cilia may be due to incomplete morphogenesis. Nevertheless, the repeated documentation of the occurrence of primary cilia in multiple vertebrate tissues and cultured cells, not only consolidated their structural model, but also supported their necessary functional significance [28,29]. The term “primary cilia” was used the first time by Sorokin (1968) when studying the ciliogenesis during pulmonary development, to designate the solitary (9+0) non-motile cilia that could be transitory, and assemble from one of the cell’s centrioles. In this same study, he described the assembly of motile cilia of the ciliated border from mature centrioles/basal bodies/kinetosomes aligned in rows beneath the apical plasma membrane. In these multiciliated cells, the basal bodies were released from fibrogranular aggregates and deuterosomes as procentrioles, prior to maturation and migration to the apical membrane [30].

Although a few researchers envisaged the involvement of primary cilia in cell signaling, most studies continued to be driven by the intent of explaining how cilia motility was generated. This led to primary cilia receiving poor attention until the 1980s. The concept that “primary, rudimentary, single, or solitary cilia” could play a role in signaling transduction from the extracellular environment to intracellular medium was again proposed by Poole et al [31]. The continued description of these cilia in multiple cell types once again supported this notion [32]. However, primary cilia were pushed to the limelight only later in the 1990s. Kosminski and Rosenbaum (1993) [33] showed that cilia present a continuous intraflagellar transport of IFT particles. This were later shown to be a microtubule-dependent transport process based on anterograde (kinesin-2), and retrograde (dynein-2) motor proteins, being essential for the assembly and maintenance of all eukaryotic cilia, motile or immotile [34,35]. In addition, Nonaka et al.; 1998 [36] showed that, in the mouse, specialized motile cilia in the embryonic node are involved in breaking the symmetry of the embryo, being required for left-right determination. However, the decisive step for the recognition of the relevance of primary cilia was the relationship between cilia anomalies and the polycystic kidney disease describe by Pazour et al.; [37]. This study showed that the loss of function of the gene encoding the IFT subunit IFT88 causes the absence of flagella in *Chlamydomonas*. Moreover, mutations in the homologous gene in the mouse, Tg737, caused death shortly after birth from polycystic kidney disease which presented shorter primary cilia.

Numerous EM studies from the 1950s to 1970s using EM showed that both the centriole/basal body, and axoneme fine structures are remarkably conserved throughout the eukaryotic phylogenetic tree. This supports the idea of these being ancient structures already present in the last eukaryotic common ancestor (LECA) [38,39,40]. Furthermore, these studies also clearly show that, despite being conserved, these structures present a certain degree of structural variability. This suggests that structurally diverse cilia evolved such as to allow cells and organisms to cope with distinct environmental challenges/signals, and functional specificities or their life styles.

**Figure 1:**
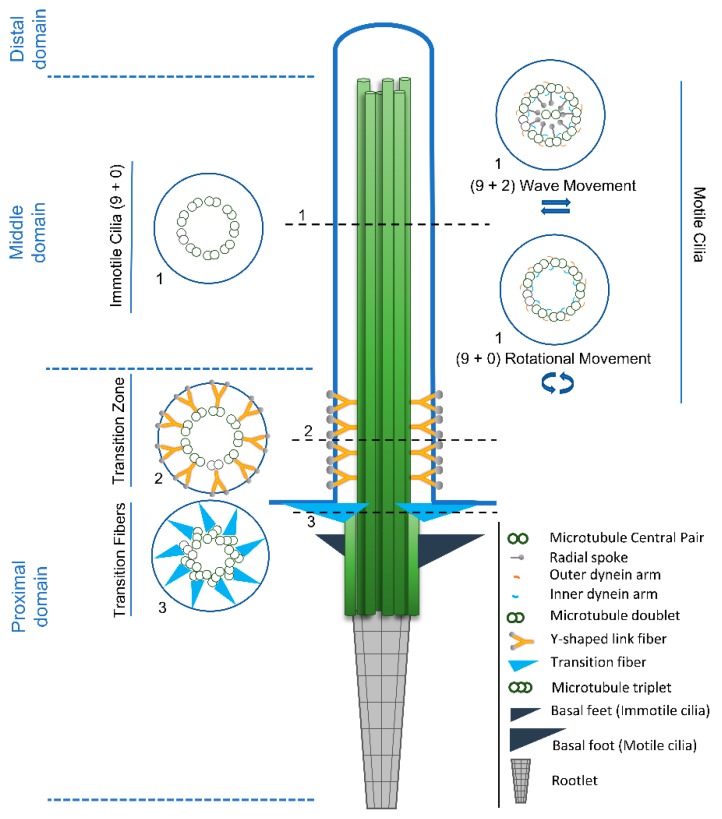
Structural comparison between motile and primary cilia. The cilia axoneme is composed of nine pairs of microtubules (green tubules) that extend from a modified centriole named basal body. Three structural and functional cilia domains can be identified, namely the distal, the middle and the proximal domains, according to their position in relation to the basal body. Cross-section 1 shows the three most common axonemal architectures: (9+0) immotile and motile cilia, with nine microtubule doublets arranged radially; (9+2) motile cilia, with nine microtubule doublets and a central pair of microtubule singlets. Motor proteins (outer dynein arms (yellow) and inner dynein arms (blue)) together with radial spokes generate the wave-like beating of the (9+2) motile cilia; (9+0) motile cilia, with nine microtubule doublets and without central pair of microtubule singlets. In (9+0) motile cilia, motor proteins (outer dynein arms (yellow) and inner dynein arms (blue)) generate a rotational movement. Cross-sections 2 and 3 respectively show, the Y-shaped links (yellow) of the transition zone and the transition fibers (light blue), that link to the membrane creating the ciliary gate. The rootlet (grey) extends from the base of the basal body towards the nucleus and appears as long striated fibers. The basal foot (motile cilia)/basal feet (immotile cilia) (dark blue) provide additional support to the cilia. In the case of motile cilia, the basal foot aligns with the cilia beating direction.

This important body of work led us to the present view on ciliary structure and functions. Thus, it is now well established that the basic ciliary structure consists of an axoneme made of nine radially-arranged stable microtubule doublets [41] templated by a centriole/basal body docked to the cell membrane, and covered by a specialized membrane domain (Figure 1). Typically, microtubule doublets are composed of a complete (13 protofilaments) A-tubule and an incomplete B-tubule (10 protofilaments) [42]. Additionally, a central pair of singlet microtubules can be present originating the (9+2) architecture. The relevance of cilia in human health and disease is also now unquestionable. Indeed, in vertebrates, most cells are ciliated, and multiple specialized cilia types fulfill sensory and motility functions critical for embryonic development and tissue homeostasis [43,44]. In simple terms, cilia can be classified as motile, promoting cell movement (e.g. protozoans and sperm cells) or fluid flow (e.g. trachea multicilated epithelia), or immotile (primary cilia) fulfilling mainly sensory/signaling functions (e.g. morphogenetic pathways including Notch, Wnt, Hippo, mTOR, PDGFR, GPCR, TGFβ and Hedgehog (Hh) signaling) [45]. However, a refined evaluation of cilia roles clearly showed that motile cilia can also mediate sensory functions, which is illustrated by the motile cilia in the respiratory and reproductive tracts of humans and other mammals [46]. Nevertheless, this idea was already latent in the early studies of protists and invertebrates showing the mechanoreception properties of their motile cilia [47].

The diverse group of signaling pathways in vertebrates that have been linked to the cilium, makes it clear that most function through primary cilia. Therefore, their loss or dysfunction is associated with developmental disorders, as a consequence of altered embryonic patterning and organogenesis. In fact, from the first work of Pazour et al. [37] to the present, the number of cilia-related diseases has significantly increased, being now designated as ciliopathies [48]. These diseases encompass variable, but often overlapping clinical features, such as blindness, obesity, cognitive impairment, skeletal anomalies, chronic respiratory problems, infertility, *situs inversus*, and polycystic kidneys. The number of ciliopathies and the number of established and candidate ciliopathy genes is still rising [49]. Interestingly, distinct mutations in one ciliopathy gene can give rise of a large spectrum of ciliopathy phenotypes [48]. Additionally, mutations affecting a number of different loci result in similar phenotypes and cause the same disease [1]. Of note, many ciliopathy mutations occur in ciliary genes that are evolutionarily conserved, and encode cilia structural components of specialized ciliary compartments such as the basal body and the transition zone. However, even though these structural components are supposed to be present in all cilia types, the mutations on their coding genes often affect cilia in a tissue-specific way. This suggests that, in addition to structural and functional cilia diversity, distinct cilia types must have specific proteomes and likely cilia-type specific roles for shared components. In support of this idea, Bettencourt-Dias and coworkers [50] have recently described specific localization patterns of 15 evolutionarily conserved ciliary proteins, as for example spindle assembly abnormal protein 6 homolog (SAS6) and centrosomal protein of 290 kDa (CEP290), at the bases of the several neuronal and sperm cilia types of *Drosophila melanogaster* [50]. Reflecting ultra-structural differences of several ciliary base-associated structures, as well as their protein composition and localization of specific proteins, this study showed that mutations in core ciliary components can generate some of the complex tissue-specific phenotypes observed in human ciliopathies. We are, however, still far from fully understanding the biogenesis and regulation of different ciliary structures and functions.

In this review we intend to give an overview of the relationship between cilia structural diversity and their functions, focusing mainly on the distal cilia tip domain and ciliary cap structures of different cilia types.

## 2. CILIA: Variations of a Basic Structure to Fulfill Myriad Functions

### 2.1. Diversity Starts at the Base: An Overview

Although the axoneme is likely the most characterized ciliary structure in multiple cilia types of many different species, cilia diversity is also reflected at the level of structures at the base (rootlets, basal feet, transition fibers, transition zone) and tip of cilia (capping structures). Concerning the ciliary base sub-structures, they can present dramatically different morphologies between organisms and even between cilia types of the same organism. In addition, they can be altogether absent, as is the case of *Caenorhabditis elegans* cilia, which lack transition fibers [51], and *Drosophila* cilia, which lack basal feet [50]. 

**Figure 2:**
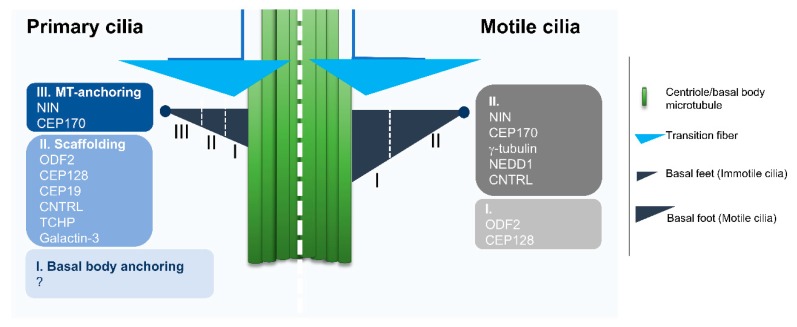
Comparison of the Structural Organization and major components of basal feet from primary and motile cilia. Primary and motile cilia basal feet present different components and domain organizations as observed by super-resolution microscopy. Primary cilia have multiple basal feet whose components are organized in three distinct domains. The most distal region (III) is characterized by the presence of Ninein (NIN) and Centrosomal protein of 170 kDa (CEP170), proteins involved in microtubule (MT) anchoring and components of the centriole sub-distal appendages. The next region (II) contains proteins that are possibly involved in the scaffolding of the basal feet, namely Outer dense fiber protein 2 (ODF2). The most proximal region (I) to the basal body was named “basal body anchoring” although there was no protein assigned to this region. Motile cilia present only one basal foot, aligned with the cilia beating direction. The domain organization is simpler than that of primary cilia, corresponding, to a certain extent to a rearrangement of Regions II and III. On the most distal region of the basal foot (II), besides the proteins in common with the basal feet of primary cilia (i.e. NIN, CEP170, Centriolin (CNTRL)), there are proteins of the γ-tubulin ring complex (γ-TURC) microtubule nucleating complex (i.e. γ-tubulin and protein NEDD1). The most proximal region (I) contains proteins that are common to the primary cilia basal feet (i.e. Centrosomal protein of 128 kDa (CEP128) and ODF2) that are most likely responsible for the scaffolding (based on proteomics and super-resolution microscopy data in [53]).

Cilia biology is intimately related to that of the centrosome, the major microtubule organizing center in animal cells. The centrosome presents two microtubule-based structures called centrioles, which are usually composed of nine microtubule triplets arranged radially. There are, however, species that present centrioles made of doublet (e.g.; certain *Drosophila* cells) or even singlet microtubules (e.g.; *C. elegans*). The two centrioles of the centrosome are asymmetric given that usually the oldest, also called mother-centriole, is decorated with sub-distal and distal appendages. These appendages have important roles in the context of cilia formation and maintenance, giving rise to basal body structures called basal feet and transition fibers, respectively (Figure 2). The mother centriole has to be converted into a basal body when ciliogenesis is triggered, and usually gives rise to primary cilia. However, motile cilia in embryonic structures like the node (mammalians) and the Kupffer’s vesicle (zebrafish) are also likely derived from the mother centriole. On the other hand, in multinucleated cells bearing multiple motile cilia, the basal bodies originate from two parallel pathways: centriole-dependent and deuterosome-dependent pathways [52].

Despite the origin of the centriole/basal body, there are several structures that can be observed at the base of most eukaryotic cilia that are poorly characterized but fulfill important roles, and reflect vertebrate cilia ultra-structural diversity (Figure 1). One of them is called the rootlet, which extends from the base of the basal body towards the nucleus and appears as long striated fibers. Rootletin has been described as the protein that organizes rootlets, but other components may yet need identification [54,55,56]. Ciliary rootlets seem to provide physical support to the ciliary structure [55], but they have been proposed to play additional roles like the transport of cargo to the ciliary base [57]. Interestingly, rootletin assembles rootlets of different lengths in different cell types, suggesting that these structures may fulfill cilia-type specific roles [54], likely in a cross-talk with other cytoskeletal components. In addition, there seem to exist inter-species diversity in ciliary rootlet structures. Indeed, Fawcett and Porter [10] recognized early, through EM studies, that the rootlets’ structure is more prominent in the cells of mollusks, less well developed in amphibia and differentially developed in mammalian. For example, ciliated epithelial cells of the rabbit trachea contain no rootlet structures, contrary to those of other mammalian species [58].

Basal feet also provide support to the cilium and acquire a prominent role in the context of motile cilia. Indeed, basal feet are critical to orient basal bodies in multiciliated cells such that there is efficient coordinated ciliary beating in the same direction, which is achieved through the basal foot role in organizing microtubules [59,60]. This is reminiscent of the role of the mother centriole sub-distal appendages, which anchor microtubules to the centrosome. In primary cilia, basal feet are important in determining the exposure of the cilium to the external environment. Indeed, the loss of sub-distal appendages/basal feet leads to the full exposure of the usually partially submerged primary cilia of human RPE-1 cells, which greatly impacts cilia-dependent signaling [61]. Typically, it has been assumed that the composition of basal feet is the same as that of the sub-distal appendages, which is supported by a few studies [61]. However, it is possible that during the mother centriole to basal body conversion the protein composition changes, something that still warrants investigation. Moreover, basal feet appear dramatically different in motile cilia. Indeed, whereas basal feet are present in multiple units in the context of primary cilia, motile cilia present one prominent basal foot (Figure 2). These two types of basal feet share components, but further studies are needed to determine their protein composition in the context of different cilia types. Additionally, it will be important to understand how the same molecules organize such different basal feet in multiple cell types. Of note, recent studies using super-resolution microscopy showed slightly different relative positions of basal feet components in primary versus motile mammalian cilia (Figure 2) [53].

The transition fibers anchor the basal body to the cell membrane. Importantly, these fibers, together with the ciliary transition zone present at the proximal end of the axoneme, function as a ciliary gate. This gate is absolutely key to determine the protein and lipid composition of the cilium compartment. In agreement, the ciliary gate is a ciliopathy hotspot with many components of the transition zone and transition fibers being coded by ciliopathy genes. Extensive reviews on the ciliary gate have been published recently and we would like to direct the readers to them [49,62]. Different transition zone architectures can be found throughout the eukaryotic tree of life or even in the same organism reflecting eukaryotic cilia functional diversity [50,63,64].

In most multiciliated cells found in the epithelia of vertebrates and other organisms, motile cilia beat in a synchronized fashion, presenting a typical (9+2) pattern. However, monociliated cells bearing (9+2) motile cilia can be found in the pronepheric duct of zebrafish [65,66]. In the case of primary/immotile cilia they are usually found alone in each cell, but there are some exceptions [67,68].

Although the (9+0) axonemal structure has been mainly associated with primary cilia this pattern is not exclusive of this cilia type. Indeed, (9+0) motile cilia are present in embryonic structures like the mouse node or the kupfer’s vesicle in the zebrafish (reviewed in [69]), and in the gametes of protists, flat worms, annelids, and eels [70]. Along the same line, the (9+2) pattern is also present in immotile cilia, as is the case of human/mouse olfactory cilia, and the cilium in mechanosensory hair cells of the inner ear. In fact, motility requires the presence of axoneme-associated structures, such as outer (ODA) and inner dynein arms (IDA), the nexin-dynein complex, which regulates the activity of the dynein arms, and the radial spokes [43,44] (see Figure 1), but not necessarily the presence of the central pair of microtubules [71]. However, there are evidences that the central apparatus is involved in determining the bending plane of cilia movement, and therefore related to the type of movement that cilia are able to promote. The (9+2) motile cilia of ciliated epithelia and sperm flagella, present a rhythmic waving or beating motion, whereas the (9+0) nodal cilia move in a rotational manner and produce a leftward fluid flow (nodal flow) [36]. The ODA and IDA are essential for ciliary motility since they generate the force that causes microtubule doublet to slide. This produces a helical beat that can be modified by the inter-doublet links and the interactions between the central pair projections and the radial spokes [72]. ODA’s function is mainly to control the ciliary beat frequency by changing the velocity of doublet sliding, without changing the beat type [73]. On the other hand, IDA, in conjunction with the radial spokes and the central pair complex, control the bend amplitude and the form of the beat [74]. In *Chlamydomonas*, ODA are composed of three dynein heavy chains while in metazoans they possess only two [42,75]. IDA are less studied, and in *Chlamydomonas*, they present seven major different heavy chain dyneins and at least three minor heavy chain dyneins [76]. The complexity of dynein arms confers, in many organisms, the ability to change their ciliary beat pattern in response to specific environmental signals. This picture may be more complex since besides the structural units of ODA and IDA are highly conserved throughout evolution [77], genomic studies show diversification of dynein genes with specific phylogenetic distribution which may be related to species specific functions [78]. Radial spokes regulate dynein activity, acting as mechano-chemical transducers between the central apparatus and the outer microtubule doublets. These T-shaped structures are composed of at least 23 distinct proteins in *Chlamydomonas* and are periodically repeated throughout the axoneme. Most organisms contain three radial spokes in each repeat unit (e.g.; *Tetrahymena*, *Trypanosoma*, sea urchins, mammals [64,79,80] while *Chlamydomonas* and *Sarcophaga* only possesses two complete and one incomplete repeat units [81,82,83,84,85]. Even though the morphology of radial spokes is conserved, their components have diverged evolutionarily. For example, *Ciona* radial spokes have only one RSP4/6 protein, whereas *Tetrahymena* has three [86].

The structural diversity in the middle domain beyond its impact in cilia motility is probably related with other cilia-specific functions. Recently, a new axonemal microtubule organization was observed in *C. elegans* cilia cephalic male (CEM) neurons, in which the cilia middle regions doublet microtubules are splayed to form complete A- and B-tubule singlets while remaining attached at their proximal and distal ends [87]. Variations to the (9+0) and (9+2) structures can be found throughout the eukaryotic tree of life. Most often, they consist either of extra structures surrounding the (9+2) axoneme (usually in sperm cells), or in the absence of one of the central microtubules [16,64,88,89] (see Table 1, for examples). This illustrates how this domain is poorly structurally/functionally characterized in diverse cilia types.

### 2.2. The Amazing Architecture Diversity of the Cilia Distal Domain: Cilia Tips Segments and Caps

In recent years, the cilium compartment started to be regarded as being composed of distinct domains that are functionally and structurally specialized (Figure 1). Here we will focus our attention of how the distal ciliary domain (the tip or tip segment) is structurally organized and may present complex capping microtubule structures (caps) (see Table 2).

The first cross sections from the transition region from the axoneme middle segment to the tip of motile cilia/flagella from a variety of organisms (e.g.; mammalian sperm [96,97]; invertebrate sperm [10,98]; gill cilia [63], ciliate protozoa [99,100]; flagellate protozoa [14]; fungal zoospores [101] and unicellular algae [102]) showed that, in general, cilia/flagella tend to narrow towards their most distal end, presenting a blunt or slightly pointed tip. This region is characterized by a progressive alteration and disorganization of the normal microtubule (9+2) pattern. Although variations can be identified, in general the peripheral microtubule doublets tend to become singlets, usually through the loss of the B-tubule. Frequently, these singlets preserve their radial organization, but their number decreases towards the distal end of the cilium/flagellum. However, this organization is completely lost when all the doublets are converted into singlets, and sometimes the central pair becomes indistinguishable. Still, in certain ciliates, as well as in *Chlamydomonas*, the central pair is observable until the end, and terminates in complex cap structures [15,103,104]. In addition, in most cilia from different origins (e.g.; rumen protozoa [99]; gill cilia [105] and *Euplotes* cilia [100,106]), amorphous electron dense material was observed between the microtubule central pair and the membrane, and the lumen of the singlets was electron-opaque just before the cilium/flagellum ends [15]. 

**Figure 3:**
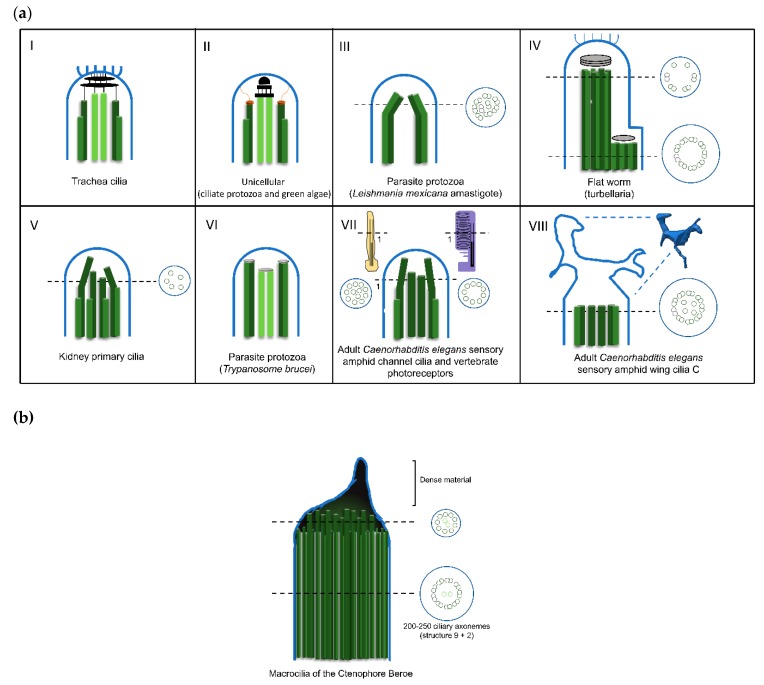
Diversity of cilia distal domain architectures. (**a**) Schematic representation of representative cilia tip structures. (I) In trachea cilia, the axoneme structure is (9+2) and at the tip the A-tubules and central pair are bound to a central cap that is attached to the ciliary crown, a structure constituted by a cluster of fibrils that project from the membrane to the outside of the cilia tip. (II) In the ciliate protozoa (*Tetrahymena*) and green alga *(Chlamydomonas*), the axoneme structure is (9+2). At the tip, the A-tubules are attached to the membrane by distal filaments that, in their proximal ends, form a plug which inserts into A-tubule lumen, while the central microtubule pair is bound to the central cap. (III). In the parasite protozoa *Leishmania mexicana* amastigote, the axoneme structure is (9+0). Distally, the 9-fold symmetry is broken by microtubule doublets progressively occupying a more central position. (IV) In the flat worm turbellarian the cilia present a typical (9+2) axonemal pattern through the main part of its length. Near the tip, this pattern changes to one where the microtubule central pair and doublets 1, 2, 3, 8 and 9 terminate in a distal cap that is attached to a ciliary crown, whereas doublets 4 to 7 end at a dense material of a proximal secondary cap. Therefore, the tip is asymmetric. (V) In the kidney primary cilia, the axoneme is (9+0) and progressively, along the axoneme, microtubule doublets suffer a displacement towards the center and the axoneme narrows towards the tip. In the distal segment, doublets are converted into singlets through the loss of the B-tubule. (VI). In the parasite protozoa *Trypanosome brucei*, the tip is (9+2) but the microtubule central pair is slightly shorter. Microtubule doublets and central pair present a dense material at the ends. (VII) In the adult *Caenorhabditis elegans* sensory amphid channel cilia (yellow) the axoneme is (9+a few singlets), and the microtubules maintain a radial organization and in vertebrate photoreceptors (purple) the axoneme is (9+0). However, both tips present only microtubule singlets. The differences can be observed at the cross section (VIII). In the adult *Caenorhabditis elegans*’ sensory amphid wing cilia C, the middle region of the axoneme contains both doublets and singlets that “splay apart” laterally and end together ~0.5 μm below the distal membrane. The tip presents a membranous fan-like structure. (**b**) Macrocilia of the ctenophore *Beroe*. Macrocilia present hundreds of ciliary axonemes with a (9+2) structure surrounded by a common membrane, with a giant capping structure. At the tip, doublet microtubules end and only singlet microtubules remain, linked together by electron-dense material. Central microtubule pairs are often still recognizable at this level. Schemes were based on: [58,92,103,109,110,111,112].

In fact, more detailed studies of thin sectioned tips of *Chlamydomonas* flagella showed that the central pair microtubules were inserted into a cap (central microtubule cap) [15,107] which was attached to the distal tip of the flagellar membrane (Table 2). The cap is composed of two plates oriented perpendicular to the long microtubule axis, that seem to be attached to each other by small filaments and a spherical bead of approximately 50 nm in diameter. At the distal end, the bead links the plates to the membrane. Also, the A-tubules of the outer doublet microtubules of the flagella terminate in a pair of filaments that were designated as distal filaments [107]. These structures are attached to the membrane and to a plug-like structure that is inserted into the tip of the A-tubules. A comparison between the *Chlamydomonas* flagella tip structures and those of *Tetrahymena* and *Aequipecten irridians* bay scallop gill cilia showed that, these present similar structures linking the ends of both central pair and outer doublet microtubules to flagellar membranes (Figure 3aII; Table 2) [104]. Moreover, in *Tetrahymena*, the central microtubule cap is bound to the ends of the two central microtubules by plug structures similar to those observed in A-tubules [108].

In the parasite protozoan *Trypanosoma*, motility is achieved through the undulating behavior of a single (9+2) flagellum. The analysis of the flagella tip of different promastigote *Trypanosomatida* species (i.e.; *Crithidia deanei*; *Herpetomonas megaseliae*, *Trypanosoma brucei* and *Leishmania major*) showed that these (9+2) flagella present a blunt end where two regions of dense material are detected (Figure 3aVI). One is essentially associated with the central pair of microtubules, whereas the other connects to the doublets [113]. Depending on the species, this dense material may show a ring shape or adopt a more disc-like structure [113]. On the other hand, the intracellular amastigote stages of *Leishmania* parasites possess a short cilium with an interesting partial structure of (9+0). In these cilia, 1 or 2 doublets are subject to displacement from the 9-ring organization throughout the axoneme, generating a variable architecture towards the cilia tip, where no organized structures are observed (Figure 3aIII) [92]. Moreover, connections between the microtubule doublets and the ciliary membrane were observed throughout the majority of the cilium length [92]. Due to the fact that these cilia are found in close contact with the membrane of the parasitophorous vacuole inside host cells, it was proposed that they act as a sensory organelle with important functions in host-parasite interactions and signaling [92].

In mammalian epithelial cilia, as the ones lining the trachea, oviducts, and bronchioles the central microtubules’ cap is amorphous in appearance. Additionally, instead of being directly linked to the cilia membrane like in protozoa, it links to a ciliary crown, a structure constituted by a cluster of fibrils emanating from the ciliary membrane at the very tip of the organelle (Figure 3aI and Table 2) [109,114,115,116]. In these cilia, the plugs of A- and central pair microtubules end in a plate of electron-dense amorphous material, and five layered discs are found in the space between the membrane and the amorphous plate [109,114,115]. The crown fibrils seem to be linked to the plates through the membrane, given that they are resistant to treatments with detergents that affect it. A similar structure, with slight differences, was described in the cilia of other vertebrates such as, chicken, rabbits, frog embryos, adult frog palates [117], Anuran seminal vesicles [118], mouse thymic cysts [116] and the haptocilia of *Acoel* turbellarians [103,119]. On the other hand, bovine tracheal cilia lack ciliary crowns, being the dense cap linked to membrane through thin filaments, resembling the way the central cap is linked to the membrane in protozoan cilia [120]. Interestingly, Dirksen and Satir (1972) [114] noticed that the cilia crown was absent from the mouse developing oviduct and respiratory tract cilia [109], appearing only when the growth is complete. This strongly suggests that the cilia tip structure varies throughout the different stages of ciliary maturation and between tissues. 

Contrary to the observations in protozoan cilia, in tracheal, oviduct, and frog palate motile cilia, the plugs are linked to the central microtubule cap instead of the membrane [120]. It is noteworthy that, in the *Tetrahymena* oral apparatus cilia, the distal filaments also link the outer doublets to the central microtubule cap [104] being an exception relatively to the remainder cilia of this organism. This indicates that the tip structures present not only a degree of structural variation from species to species, between different tissues and developmental stages of an organism, but also within a single-cell organism. 

Asymmetrical cap structures were found in certain amphibian cilia, as well as in *Acoel* turbellarians (flatworms) (Figure 3aIV; Table 2) [119,121]. For example, palate cilia in the frog *Bombina orientalis* end in one large cap, linked to the membrane and to doublet microtubules number 4 to 7, and a smaller cap that is connected to doublets number 1, 2, 3, 8, and 9 and the two central microtubules. A plug structure inserted into the lumen of each microtubule attaches the A-microtubules to the caps [121]. In the case of *Acoel* turbellarians, the adhesive plate on the ventral and posterior tail surface possesses cilia with a typical (9+2) axonemal pattern through the main part of its length. However, near the tip the microtubule central pair and doublets 1, 2, 3, 8 and 9 terminate in a distal cap, whereas doublets 4 to 7 end at a dense material of a proximal secondary cap [103,119]. The distal cap has 2 to 4 laminar structures, and possesses attached filaments that resemble the fibrils of the ciliary crown of the mammalian tracheal cilia cap. Interestingly, some of the doublets end as a single A-microtubule, whereas for others, the A- and B-microtubules terminate in the cap. These cilia are motile, display slow, irregular beating with an irregular bending and most probably possess an adhesion role, allowing the animal to stick to the substrate [103]. Noteworthy is the fact that in both organisms the same groups of microtubules are linked to distinct caps but the orientation of the asymmetric structure, in respect to the cilia stroke are different [121].

Different cap structures, essentially composed of amorphous electron-dense material distributed around the distal ends of the doublet and central microtubules, were identified in cilia from different organisms. The lips of the ctenophore *Beroe* possess multiple rows of motile ciliary axonemes surrounded by a common membrane, and these are thought to help the organism spread its lips over preys, pushing them into the stomach cavity [110]. These multiple axonemes present a giant capping structure at the tip, formed by extensions of the A- and central-pair microtubules, which are bound together by amorphous material (Figure 3b; Table 2) [110]. The sensory cilium of the chordotonal sensillum in the American cockroach antenna pedicel, possesses an axoneme with a (9+0) pattern throughout its length. At its dilated the tip, the doublets become singlets and terminate in an electron-dense spheroid [123]. It was proposed that these cilia may be involved in mechanoelectric transduction, when vibrations are applied to the sensillum [123]. Additionally, dense material was observed at the distal ends of the axonemal microtubules in stiff non-motile sensory cilia of the bivalvia *Lima hians* [122].

Concerning the ultrastructure of primary cilia distal tips, not much data is available, but their tips seem to lack cap structures or even electron-dense material (Figure 3aV) [124]. In cultured kidney IMCD3 cells the doublet microtubules suffer a displacement, distorting the (9+0) arrangement towards the tip. Moreover, these cilia narrow towards the tip where a few doublets and singlets are still detected [92]. However, in many of these cilia, the organization pattern of the microtubule doublets can be extremely variable along their length, and many of them present connections between the doublets and the ciliary membrane throughout the axoneme. In certain cases, these structural details associated with their specialized functions may overcome the necessity to have a cap structure. 

This analysis of how different cilia organize their distal segment suggests that during evolution, eukaryotic cilia have acquired or modified ciliary capping structures in order to fulfill specific roles in different cilia types and this is reflected by their multiple structural modalities. Even single cell organisms present different functional cilia/flagella and these multiple ciliary tip-organizations and associated structures seem to be related to specific ciliary roles. In the case of motile cilia, it has been proposed that those involved in cell propulsion, for example in ciliates and flagellates, present tip cap structures that evolved to allow beating and locomotion in water. These caps will probably help to maintain cilia structure integrity continuously under mechanical stress caused by beating, still accommodating the possibility of dynamic regulation of cilia length in response to stimuli. On the other hand, the specialized structures (ciliary crown) found in the majority of vertebrate ciliated epithelia (Table 2), likely allow for efficient interaction with ovules and movement of viscous mucus and lubricants required for tissue homeostasis. These processes, compared to cellular movement, will also require different force generation. The crown fibrils probably increase adhesion efficiency creating a larger surface and allowing a better adaptation to interaction with random shapes. Still, exceptions, like that of bovines limit this view. Interestingly, similar structures are found in cilia of *Acoel* turbellarians, which appear to be involved in sticking the organism to a substrate. It is also possible that these cilia structures may have evolved from early organisms for which cilia adhesion properties to particles/preys or substrates would favor fitness. It is tempting to hypothesize that the crown fibrils may be able to transduce signals from adhesion interactions to the cilia tip. If this is true these signals could trigger local modifications in microtubule dynamics and increase/decrease in a few nanometers the length of the distal segment which would adapt stroke to the physical/chemical features of the media. Supporting this idea is the fact that motor cilia are able to change size in response to environmental stimuli [125]. The biochemical characterization of distinct cilia types in different organisms and tissues will definitely help elucidate the functions of these diverse ciliary capping structures. However, despite the limited information on the molecular composition of the ciliary surface, it is interesting that cilia tips present adhesive properties illustrated by the ability of virus and bacteria to adhere to cilia [126]. In fact, cationic ferritin binds preferentially to cilia crown, showing the presence of anionic sites in these localizations [127].

The asymmetry of some cap structures was proposed to be related with the definition of the cilia stroke orientation, but no constant pattern was found in the different examples described so far. In the case of the *Acoel* turbellarians the accentuated asymmetry creates a small thinner segment in the tip that will also allow a better interaction with substrates with random shapes which may create opportunities to better sense the substrate and adhere. In the case of frog palate cilia asymmetry is probably important to help define the direction of the stroke. These cilia propel mucus and particles in it over of the oro-pharynx epithelium in the posterior direction and down the oesophagus [128]. Interestingly, they are shorter than cilia generating water propulsion and present relatively small difference in height between the effective stroke and the recovery stroke which seems to help the mucus movement. Additionally, these cilia do not present a high coordination showing intermittent activity, which suggests that cilia motility is regulated by reflex control dependent upon mechanical stimulation. Dentler [58] suggested that cap structures may cause modification in the patterns of microtubule sliding. This modification would cause a twist along the axoneme or create an effective stroke with a nonplanar bend. Alternatively, the microtubules may stretch or contract during sliding with the purpose of accommodating the restriction of sliding by the cap. Therefore, different cap structures may contribute to different cilia bending patterns.

Finally, sensory cilia seem not to have complex structures at their tips but in certain cases amorphous material can be observed. Whatever cilia are essentially motile or sensory the analysis of their distal cilia compartments shows that in the majority of the cases the microtubule pattern of the axoneme of nine doublets tends to be progressively converted in singlets by disappearance of the B-tubules. Thus, most cilia before ending in a cap+plugs or dense electron material or not showing any visible structure present a short segment of singlets.

### 2.3. The Distal Domain of Cilia: Components and Functions

The existence of cap structures displaying complex architectures in several cilia types, poses several questions concerning the structural/functional compatibility of these structures with the events known to occur at the cilium distal domain. For example, studies in *Chlamydomonas* have shown that in growing axonemes the precursor proteins, including tubulin heterodimers, are transported by the IFT machinery to the tip where the assembly occurs [129,130,131,132]. *Chlamydomonas* flagellar resorption also seems to occur by disassembly of axonemal microtubules from the tip [107,133]. Furthermore, the central microtubule cap is maintained linked to the microtubule central pair both during cilia recovery and disassembly (resorption) [107]. Although the distal filaments linked to the plugs seem to be more loosely attached to the outer doublet microtubules, these structures stay linked to the microtubules during their elongation [104,107]. In tracheal cilia, the ciliary crown is preserved on cilia even when the cilia membrane is removed using triton X-100, which strongly supports these structures being tightly attached to the microtubules [109]. This suggests that even though caps are clearly structurally different between organisms such as *Chlamydomonas* and vertebrates, these may have similar composition and functional properties.

As previously mentioned, in *Chlamydomonas* mature and capped flagella, tubulin assembles and disassembles continuously at their distal end, indicating that the tips are dynamic structures [134]. Also, in the *C. elegans* sensory cilium, microtubule plus ends lying at the distal (A-tubule) and middle (B-tubule) segment tips exchange tubulin subunits and these localized dynamics are necessary to maintain proper axoneme structure [135]. Supporting this view is the observation that *Tetrahymena* tubulin-knockout cells, at ∼26 h post-mating, lacked most microtubules and had a dramatically reduced cilia number. Additionally, among the remaining (pre-existing) cilia, many had splayed tips, likely due to the lack of new tubulin subunits’ addition, of resulting in tip instability [136].

Moreover, the dynamics of cilia distal segments allows changing the length of this ciliary domain in response to external stimuli, like the absence of sensory signal transduction, and hypo- or hyper-osmotic challenges [111,137]. Interestingly, the tubulin diffusion rate is slower at the cilia tip than at other ciliary regions, which suggests that tubulin interacts with other proteins at the cilia cap [138]. Therefore, these structures may regulate tubulin turn-over, as well as that of other cilia components at the tip domain. 

The distal compartment is also where IFT particle trains, transported by kinesin II in most organisms [139,140,141], release their cargo. In addition, they undergo a reorganization process that includes the split apart of the IFT complexes and their mixing with other complex components from other IFT trains. This includes the switch of motor protein so that the trains are transported to the ciliary base by dynein 2 [142,143,144,145]. Therefore, the tip, in parallel with the ciliary base, is one of the critical points in the regulation of IFT association with their cargo. This regulation will be in a constant interplay with the mechanisms involved in cell cycle regulation and cell responses to environmental changes [146].

How the complex cap structures accommodate the dynamic behavior of the overall cilia tip region is even more puzzling, knowing that they assemble early during cilia recovery after deciliation, at least in *Chlamydomonas*, *Tetrahymena*, *Trypanosoma* and amphibian ciliated epithelia [104,112,147] while the cilium is still growing. In addition, and most importantly, some axonemal components, such as the axonemal dynein 22S complex [148] and the radial spoke complexes are preassembled in the cell body before their transport and integration into the growing cilia [149]. 

**Figure 4:**
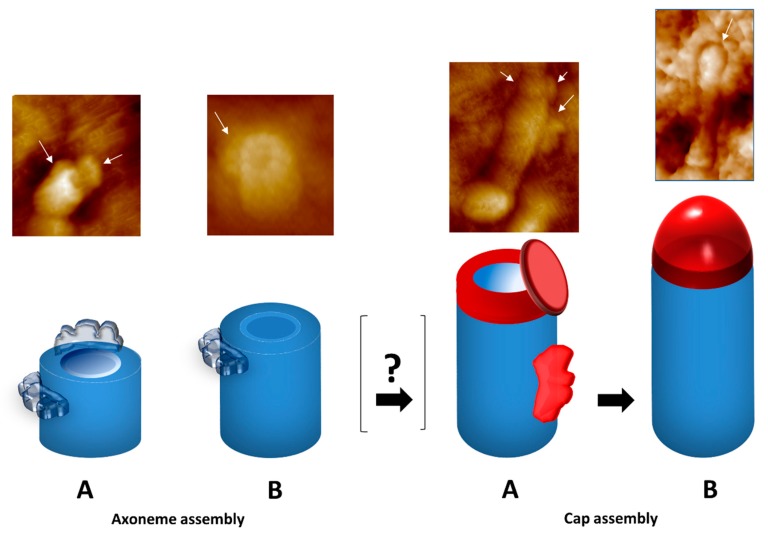
Several stages of *Tetrahymena* cilia assembly model. Axoneme assembly: (A) Three-fold structures present at the transition region and axonemes (210–460 nm) of growing *Tetrahymena* cilia. It was speculated that the three-fold structures function like ring sections that will progressively assemble transversally, originating a full ring (9-fold structure) contributing to the axonemal growth. Cap assembly: (A) A collar-like shape is assembled in the axonemal distal tip, shown in red, providing an incomplete cap to the cilium. The other components of the cap are larger structures that are visualized near the edge of the cilium tip (A); (B) All cap components are assembled and finally originate the full mature cap with a dome shape. In blue, the axoneme wall; in transparent dark blue the three-fold structures. The different components involved in cap assembly are shown in red. The AFM topographic images representative of the different steps of cilia and cap assembly are shown at the top of the schematic drawing. Reprinted with permission from Reference [150].

How the complex cap structures are assembled is almost unknown. Studies in *Tetrahymena* and frog ciliated epithelia strongly support the idea that the assembly of the cap is a multi-step process, in which structures of different sizes and shapes are brought together before the axoneme appears fully capped, and are recruited early during cilia assembly (Figure 4) [147,150]. Atomic Force Microscopy (AFM) in tapping mode, with resolution at the nanometer range, and with minimum sample manipulation, was used to follow the first steps of *Tetrahymena* cilia assembly [150]. This method allowed the observation that this process requires the transient assembly of structures, composed of three components that are placed asymmetrically on an early elongating axoneme wall (Figure 4) [150]. These structures are visible in small uncapped axonemes ranging from 210–460 nm in height. In these small axonemes, the microtubule central pair was never observed, which is probably due to the fact that these singlet microtubules have delayed polymerization compared to those forming the outer doublets, and thus are not visible on the growing tip. In fact, this seems to be the case, as a more recent study showed that the central pair, capped by the ciliary tip complex, can be over a micrometer shorter than the B-tubules [151]. Growing axonemes with ∼1 μm of length present a collar-like structure of 200 nm in thickness that is probably an incomplete cap at their distal tips. The complete cap seems to be assembled afterwards, upon the arrival of additional structures also visible on the small cilium wall (Figure 4). Similarly, Portman et al. [147] described that, in the cilia of palate epithelial cells of the frog *Bombina*, the cap assembly process begins with the formation of a disk-shaped plate during the first µm of growth. In fact, electron-dense material, similar in appearance to the capping structures, was observed between the microtubule tips and the ciliary membrane in small cilia of 500 nm. The *Tetrahymena* ciliary cap seems to be fully formed by the time cilia reach ∼1.5 µm in length, whereas in the frog it seems to be completed after the first 2 μm, when they become indistinguishable from the caps of fully mature cilia.

The role of tip structures isolated from rabbit trachea was investigated in vitro, and their binding to the ciliary microtubules is sufficient to block the addition of tubulin subunits [152]. This led to the proposal, by Dentler and LeCluyse, that the regulation of axoneme microtubule polymerization depends on how caps and plugs associate with their tips [152]. Furthermore, these authors proposed a mechanism where a momentary release of the plugs from the microtubule walls would allow tubulin addition to the microtubule growing tips. This is supported by the observation that the attachment of caps and plugs to microtubules seems to vary throughout the process of *Chlamydomonas* flagella regeneration. During this process, the plugs seem to become more tightly attached to A-tubules as the flagellum reaches its full length [104], which may inhibit microtubule polymerization. Although the cap prevents the addition of tubulin heterodimers to central microtubules in vitro, uncapped microtubules present in the same preparation were able to nucleate and polymerize [106]. This led Dentler to propose that the central microtubule cap is a nucleation center for the assembly of these microtubules that grow by their proximal ends [104]. This suggests that tip structures may regulate microtubule assembly and cilia length during cilia/flagella regeneration. In *Tetrahymena* reciliation studies, small cilia between 1.5 µm (with a complete cap) and the full-length of 7 μm (average size of mature cilia) showing different stages of cap assembly were rarely observed. In contrast, smaller growing axonemes were frequently detected, which suggests that *Tetrahymena* cilia present a first stage of growing that occurs at a relatively slower pace until they reach 1.0 ∼ 1.5 µm, which is coincident with cap assembly. The capped axonemes then lengthen at a faster rate, which strongly supports the idea that the cap modifies the axoneme microtubule polymerization rate. This hypothesis is fully supported by the recent work that shows that *Tetrahymena* cilia present fast and slow growth phases, and that the slow elongation phase is a period of continued maturation process of the tip region [151]. As suggested by Portman et al. [147] it is conceivable that during cilia biogenesis, the assembly of capping structures may structurally stabilize the growing microtubules by maintaining them straight, which would facilitate their polymerization and/or assist in the correct placement of proteins like tubulin, dyneins and radial spokes. 

In *C. elegans*, IFT transport delivers distinct tubulin isotypes to the ends of axonemal microtubules of sensory cilia, where they become differentially localized. For example, the β-tubulin isotype TBB-4, localizes all along the cilium, whereas the α −tubulin TBA-5 isotype concentrates at the distal cilia singlets [135]. Similarly, cilia-specific tubulin isoforms were found in mammals [153,154]. The occurrence of specific tubulin isotypes at the cilia tip may contribute to confer specific properties to the microtubules in this ciliary domain, which in turn will be recognized by specific structural/regulatory proteins that travel to and/or are resident there. On the other hand, tubulin functional diversity is also conferred by different post-translational modifications. Thus, it is possible that caps promote post-translational modifications of microtubules, creating codes distinct from those of the microtubules in the middle axoneme section. This idea is supported by the fact that some ciliary post-translational modifications such as glycylation in ependymal motile cilia are only detected after the cilia reaches a certain length, which may be related to maturation and full cap assembly [155].

The structural and functional dynamics of the cilia tip is probably associated with the requirement of specific cilia tubulin isotypes and post-translational isoforms in order to achieve correct microtubule spatial organization, with strictly regulated microtubule dynamics and associated turn-over. Furthermore, this should maintain a confined space in close association with the cilia membrane, and keep the ability to respond to external stimuli. In this scenario, the cilia tip may require specific microtubule regulators that will operate at this domain in association with caps structures, when present.

### 2.4. What Can We Learn about the Functions of the Distal Domain from Proteins Residing There?

Compared with other ciliary domains, not much is known about the structural proteins of the tip, probably because the lability of these structures hinders their biochemical characterization [107,156,157]. Concerning the microtubule capping structures, the first cap component to be identified was a Tetrahymena protein of 97 kDa that was detected using autoantibodies of patients with CREST (Calcinosis/Raynaud’s phenomenon/Esophageal dysmotility/Sclerodactyly/Telangiectasis), as kinetochores markers [157]. These first results strongly supported the hypothesis that caps and kinetochores may share similar regulatory mechanisms for microtubule polymerization/depolymerization. Still, the identity of the *Tetrahymena* protein(s) recognized by these sera remains unknown. From this study to the present, a number of proteins that accumulate/reside at the ciliary tip have been identified and can be clustered in distinct functional groups.

#### 2.4.1. Molecular Chaperones and Cilia Distal Domain Dynamics

Several molecular chaperones have been identified in the cilium compartment, some accumulating at the tip. For example, the molecular chaperone heat shock 70 kDa protein (Hsp70) is present in *Chlamydomonas* flagella, concentrating at the tip [158]. This protein is released from axonemes at the same ionic conditions that cause the release of capping structures, which led to the suggestion that it may participate in the transport of tubulin heterodimers and/or the assembly of axonemal microtubules at the level of the cap. Worth noting, Hsp70 was identified as one of the components of a 17S complex of inner dynein arms in *Chlamydomonas* [159]. In addition, subunits of the cytosolic chaperonin containing TCP-1 (CCT), which folds a wide range of newly synthesized proteins including tubulin (alpha, beta and gamma) and actin, are present in cilia and accumulate at the tip [160]. In *Tetrahymena*, after the first 15 min of reciliation (axonemes do not contain cap structures), a decreased labeling of CCT-subunits is observed at the top of the growing axoneme. On the other hand, CCTα/TCP-1 clearly accumulates at the tips of mature cilia [136,160]. Cells lacking CCTα/TCP-1 and CCTδ subunits are unable to recover their cilia, and reduced levels of CCTα/TCP-1 present short cilia with splayed tips or with abnormal spotted tubulin staining pattern at the distal domain [136]. In agreement, in sea urchin embryonic and rabbit tracheal cilia CCTα/TCP-1 was progressively detected in the final stages of sea urchin cilia regeneration [161]. Together, these data suggest that CCT-subunits may be associated with the distal ends of microtubules and caps or even be components of the latter. Recently, it was shown that the anterograde IFT motor kinesin-17 and α-tubulin can diffuse in the axoneme lumen [162]. This pool of diffusing tubulin likely results from axonemal microtubule depolymerization [163]. Furthermore, it can possibly be used in cilia growth/turn-over, with chaperones playing a role in this process. Therefore, the concentration of molecular chaperones at cilia tips reinforces the idea that the cap may assist the assembly of the axonemal structure, and/or is involved in quality control during the turn-over of ciliary proteins, including tubulin.

#### 2.4.2. Regulators of Microtubule Dynamics at the Cilium Distal Domain

##### +TIPs and Related Proteins

One group of proteins that play important roles at the distal compartment is that of microtubule plus-end-tracking proteins (+TIPs), which accumulate at the growing ends (+) of microtubules to regulate their dynamics, and mediate interactions with other cellular components. End-binding proteins (EB) function mainly as scaffolding proteins at the microtubule plus-end, where they form a hub for other +TIPs to associate with, and thereby regulate local protein composition and microtubule dynamics. In *Chlamydomonas*, the EB1 (CrEB1) orthologue was found to localize to the tip of full-length, growing, and shortening flagella, and to the proximal part of basal bodies. Similarly, EB1 is also present at *Giardia* flagellar tips [164]. EB1 depletion in *Chlamydomonas* perturbs flagellar assembly and the accumulation of IFT complexes at the tip [165]. Indeed, CrEB1 seems to participate in IFT particle turnaround at the flagellum tip, interacting with IFT172, which controls flagellar assembly/disassembly there [166]. Moreover, EB3 has been shown to localize at the tip of *Xenopus* and human motile cilia [167], and affects the formation of centriole-associated rootlet filaments [168,169]. EB3, but not EB1, was also found in the outer segment of isolated mouse photoreceptors [170], suggesting that it localizes to the tip of specific cilia types. Live imaging analysis of human RPE-1 cells expressing GFP-EB3 [169] showed that it accumulates at the primary cilium tip and moves dynamically within the organelle. In human cells, EB1 and EB3 are required for primary cilium assembly, and cells depleted of these proteins have problems in microtubule anchoring to the centrosome/basal body. Additionally, EB1 or EB3-depleted cells form abnormally short cilia stumps surrounded by vesicles, indicating defective vesicle trafficking [168]. 

Another protein localizing at the ciliary tip is the microtubule-associated protein l CLAMP (Calponin homology and microtubule-associated protein, also called spef1). This protein contains a Calponin Homology (CH) domain similar to that of EB proteins and stabilizes microtubules, promoting bundling [171]. Moreover, it presents protein fuzzy homolog (Fuz) dependent-accumulation at cilia tips [172]. Fuz is an effector of the planar cell polarity pathway (PCP) that seems able to differentiate between retrograde and anterograde IFT complexes. Fuz knockdown in vertebrate multiciliated cells leads to the accumulation of IFT-B proteins and alteration of normal IFT dynamics, decreased axoneme length and shortening of the CLAMP enrichment region, which perturbs the distal ends of axonemes [167]. As in the case of EB proteins, these results show that the alteration of axonemal microtubule dynamics affects IFT rates, and points to a close relationship between this regulation and PCP.

The members of the domain array-containing protein family, ch-TOG, are key regulators of cytoplasmic microtubule dynamics also present at cilia tips [173]. In mammalian cells, Crescerin 1 accumulates at the basal body and tip of the primary cilium, as well as, along the lattice of cytoplasmic microtubules when overexpressed [174]. The tumor overexpressed gene (TOG) domain of Crescerin 1 possesses inherent microtubule-binding activity and promotes microtubule polymerization in vitro. In *C. elegans*, CHE-12/Crescerin is necessary for proper cilia development and its expression is restricted to a small set of sensory neurons that have a simple rod-shaped cilium [174]. In *Tetrahymena*, CHE-12/Crescerin accumulates at the tip, near the B-tubule [175], and its loss of function leads to cells with less and shorter cilia. Given its localization and ability to promote microtubule polymerization in vitro, CHE-12/Crescerin most probably promotes the elongation of B-tubules [175]. In fact, in *Tetrahymena* CHE-12/Crescerin knockout cells, the ciliary distal segment was longer and without microtubules, possibly due to shortening of all microtubules, being the B-tubules the most affected [175]. These tip alterations neither affected cilia beat waveform nor its frequency. 

Another protein that acts at B-tubules’ + ends is LisH domain-containing protein ARMC9 (ARMC9), mutations in which cause Joubert syndrome in humans, and ciliopathy phenotypes in zebrafish [176,177]. When stably expressed in mammalian IMCD3 or NIH-3T3 cells, ARMC9 localizes at the ciliary proximal region, and accumulates at the tip upon Hh signaling [178]. Supporting a role in this Hh signaling, after activation of the pathway, NIH-3T3 Armc9-mutant cells show less ciliary accumulation of GLI2 and GLI3 (Glioma-transcription factors GLI), but not the Smoothened (SMO) receptor. These data strongly suggest that ARMC9 participates in the trafficking and/or retention of GLI proteins at the ciliary tip, being a signaling factor [178]. *Tetrahymena* ARMC9-KO cells show cilia with increased length [175]. However, whereas central pair region was unaffected, the entire distal segment and A-tubule region was reduced. Indeed, the B-tubules were markedly long compared to the A-tubules. On the other hand, the overexpression of GFP-ARMC9B causes a longer distal segment. Together, these data suggest that ARMC9 activity is required to shorten the B-tubules. In *Tetrahymena*, the cilia of double CHE12/Crescerin and ARMC9 knockout cells show normal length but severely compromised functions [175]. Accordingly, the authors suggested that ARMC9 and CHE12/Crescerin act in two competing pathways and that the cilia tip organization, instead of cilia length, is the cause of abnormal ciliary functions when the two proteins are removed [175]. Given that both proteins act on the ends of B-tubules, regulating their elongation and shortening respectively [175], it is plausible that their balanced activity is required for correct cilia length establishment.

*Chlamydomonas* FAP256 (homologue of human centrosomal protein of 104 kDa (CEP104)) localizes at the tips of both the microtubule central pair and A-tubules during flagellar assembly and disassembly [179]. In human non-dividing cells, CEP104 localizes at the distal ends of both centrioles until the mother centriole is converted into a basal body. Then, it becomes localized at the tip of the cilium. Human tracheal cilia also present CEP104 at the tip [179]. Collectively, these results suggest that CEP104 localization does not rely on the presence of cap structures. Recently, Louka et al. [175] showed that FAP256/CEP104 is also enriched in the distal domain of Tetrahymena cilia. In human, *Chlamydomonas* and *Tetrahymena* cells, the loss of the FAP256/CEP104 results in structural malformations at ciliary tips. In addition, flagella shortening was observed in the algae. Human and *Tetrahymena* FAP256/CEP104-depleted cells also show less primary and motile cilia, respectively [175,179]. In *Chlamydomonas*, as a consequence of CEP104 depletion, the central cap plate is lost and the outer-doublets plus central-pair microtubules extend to the overlying membrane. In addition, the radial spokes spread from the A-tubules and are visible almost to the very tip of the cilium which appears blunt and more rounded, losing the characteristic cone-shaped bulge [179]. Notably, *Tetrahymena* FAP256/CEP104-KO cells do not show significant alterations in cilia length, but present a reduction in the length of the distal segment due to the shortening of both the A-tubule and the central pair regions. In both protozoa, the shortening of the central pair region seems to be due to the loss of the central microtubule pair cap, the end plate in *Chlamydomonas* and the bead structure in *Tetrahymena* [175,179]. The studies in *Tetrahymena* strongly support the idea that FAP256/CEP104 elongates the A-tubules and stabilizes the central microtubule cap, and this may also explain the blunt tip phenotype in *Chlamydomonas*.

In mammalians, CEP104 is a multidomain protein that was identified as an EB protein-dependent microtubule plus-end-tracking protein (+TIP). In addition to interacting with EB1 and tubulin through a conserved TOG domain [180], CEP104 interacts with a number of other proteins, like centriolar coiled-coil protein of 110 kDa (CP110) and centrosomal protein of 97 kDa (Cep97), which suppress cilia growth [181]. CEP104 may regulate cilia assembly by interacting with the CP110-Cep97 complex, participating in its removal from the mother centriole distal end, allowing for cilia growth. This CEP104 role may rely on EB1 binding to the dynamic microtubule ends [179,180,181]. Taking all these results into consideration, we can think that CEP104 may control cilia length and tip organization by regulating microtubule dynamics between the singlets and the central pair. In *Tetrahymena*, the overexpression of FAP256A/CEP104 originates long fibers suggest this protein is able to oligomerize on the microtubule surface, like the mitotic plus end-tracking proteins, kinetochore protein NDC80 homolog (NDC80) and DASH complex subunit DAM1 (Dam1) [182,183]. This led Louka et al. [175] to propose that the oligomerization of FAP256/CEP104 contributes to its preferential recognition of the ends of complete microtubules (A-tubules and central pair). The *Tetrahymena* data also indicates that correct microtubule dynamics at the cilia tip are related to the cap structure maintenance/assembly. Considering the role of the protein at the distal region of the mother centriole, where it allows ciliogenesis, it is plausible that FAP256/CEP104 also plays a role as a platform for the assembly of the cap, mediating the interaction between specific components and the axonemal microtubules’ plus ends. This cross-talk may also be mediated by EBs proteins.

##### Kinesin Proteins

Other tip-binding proteins, including distinct kinesins, also reside at the ciliary distal domain. Class 13 kinesins are targeted to both microtubules ends, either directly [184] or by diffusing along microtubules [185]. At microtubule ends they cause a structural conformational change that promotes their disassembly in an ATP dependent manner [186]. Therefore, these kinesins are unique microtubule depolymerizing enzymes that regulate, for example, spindle microtubule dynamics and kinetochore-microtubule attachments [187]. Interestingly, members of this kinesin class such as mitotic centromere-associated kinesin (MCAK) associate with both EB1 and EB3, co-localize with EB1 at microtubule plus ends [188,189], and have ciliary roles not always conserved between species. For example, in *Giardia intestinalis* kinesin-13 localizes with EB1 at flagella tips and a mutant strain for its coding gene shows a dramatic increase in flagella length [157]. In agreement, in *Leishmania major*, kinesin-13 overexpression causes flagellar shortening whereas its knockdown yields long flagella [190]. These results thus suggest that kinesin-13 regulates flagellar length dynamics in both protozoan parasites. In *Chlamydomonas*, on the other hand, kinesin-13 (CrKinesin-13) is transported to the tip of flagella by IFT, plays a role during flagellar regeneration and shortening [191], but does not seem to be involved in flagellar length control. Likewise, in *Trypanosome brucei* neither kinesin-13 gene deletion, nor its overexpression, cause significant alterations on the flagellum length, or the growth rate of the new one [192]. This lack of functional conservation in what concerns flagella length may be related to the different flagellar tips presented by these different protozoa species. Alternatively, or in addition to, kinesin 13 may be part of different molecular networks in the different organisms, which will condition its function in each specific context. In fact, despite having potent microtubule depolymerization activity, these proteins are able to accumulate in the plus ends of microtubules [193] suggesting that the microtubule-depolymerizing activity must be regulated at this site. Therefore, cilia length control may require different players probably acting through distinct mechanisms, reflecting axoneme and cap diverse structures, and cilia/flagella specific functions. Supporting this idea is the fact that other kinesins seem to be involved in cilia length control, e.g.; kinesin-like protein KIF19 (KIF19A). This member of the kinesin 8 class possesses both microtubule-based plus-end directed motility and microtubule depolymerizing activity in a length-dependent manner [194,195]. In the mouse, kinesin KIF19A localizes to ciliary tips and depolymerizes microtubules mainly from their plus ends, being a key regulator of motile cilia length [196]. Moreover, the knockout of the kif19A gene causes ciliary phenotypes such as hydrocephalus and female infertility [196].

The role of distinct kinesin families in cilia/length control by destabilizing microtubules ends, either at motile or primary cilia tips, is emerging as a common pattern of function. Indeed, Pedersen and Akhmanova proposed that the recruitment of negative regulators of microtubule polymerization might be a general feature of the length control mechanism of the otherwise strongly stabilized axonemal microtubules [197]. However, the cilia length determination mechanisms may probably be more complex, since microtubule destabilization seems to be also required to maintain sensory/signaling functions at the cilia tip.

In mammalian primary cilia, kinesin-like protein KIF7 (KIF7), a member of the kinesin-4 class, organizes the ciliary compartment and regulates the activity of GLI transcription factors, being required for correct Hh signaling [198]. KIF7 displays no motility, is unable to diffuse along microtubules [199], localizes to the base of primary cilium in the absence of Hh signaling, and shows an Hh depending-accumulation at the ciliary tip [198]. As previously mentioned, members of the Hh pathway, like Gli proteins and Sufu (Suppressor of fused, negative regulator of Hh), also accumulate at cilia tips [200]. He et al. [199] showed that the N-terminal fragment of KIF7 containing the motor domain and the first coiled-coil segment of the protein binds the plus-ends of growing microtubules in vitro. In fact, it preferentially associates with GTP-bound tubulin, causing the reduction in microtubule growth rate, and increasing the frequency of microtubule catastrophe. In the absence of KIF7, cilia are longer and unstable, showing a twisted morphology which suggests defects in the axoneme structure [199]. Therefore, KIF7 seems to inhibit the polymerization of microtubule plus-ends and control the length and structure of primary cilia, which is required for the correct localization of Hh signaling complexes. Indeed, in the absence of KIF7, the Hh components Gli and Sufu do not localize at the tip compartment being instead present along the axoneme, causing signaling deregulation [199]. KIF7 might also contribute to the organization of Hh signaling complexes through direct interactions with Hh pathway components at the cilia tip (for review [197]).

Intriguing is the mechanism by which immotile KIF7 accumulates at the cilia tip. Liprin-α1 (PPFIA1) and the protein phosphatase PP2A are KIF7-interacting proteins important for the trafficking of KIF7 and Gli proteins to the tips of cilia, and the activation of the transcriptional activity of Hh target genes [201]. The KIF7 phosphorylation state establishes its localization. The concerted action of liprin-α1 (PPFIA1) and phosphatase PP2A promotes KIF7 dephosphorylation and subsequent localization at primary cilia tips, where the kinesin determines the outcome of Hh signaling [201]. This regulatory picture may be more intricate since the small GTPase Arl3 and its regulatory GTPase activating protein (GAP), Retinitis Pigmentosa 2 (RP2), also interact with KIF7. These proteins also mediate the localization of (KIF17), a homodimeric Kinesin-2 class motor, to cilia tips [202]. Depletion of Arl3 or RP2 by siRNA in human cells causes a decrease in KIF7 and KIF17 levels at the tip. Both Arl3 and RP2 are able to interact with KIF7 and KIF17, suggesting they play a role in the localization of these kinesins [202]. KIF7 functions upstream of KIF17 since its depletion of in RPE-1 cells causes a decrease in KIF17 levels at the tip, but the reverse is not true [202]. Consequently, KIF7 is required for correct KIF17 localization to the tip, either directly or by shaping this ciliary compartment [199]. Similar to what occurs with KIF7, the loss of KIF17 alters cilia length but has no impact in GLI localization. KIF17 localizes at the plus ends of growing microtubules where it promotes tubulin acetylation, which requires the interaction with EB1 [203]. Additionally, mouse mutants of KIF7 present altered patterns of tubulin acetylation and polyglutamylation along the axoneme. This suggests that KIF7 promotes the stability of axoneme microtubules and likely their association with microtubule-binding proteins [199].

These examples show that the spatial organization of cilia tips is dependent on the functional relationships between different kinesins. Most likely, the continued reorganization/shaping of this compartment is required for correct signaling. It is plausible that these dynamics rely on the turn-over, probably mediated by molecular chaperones, of tubulin isotypes/isoforms. This may be a requisite for the recruitment/organization of other ciliary molecules at the tip, and consequently for regulating signaling responses. Kinesins’ concerted roles may create specific environments that will fine-tune ciliary functions. For example, in *C. elegans* neurons, the morphologically diverse sensory cilia are related to the ability of the animal to sense different stimuli. Amphid channel cilia show an unbranched cylindrical morphology and are dedicated to sensing hydrophilic molecules and high osmolarity (for tip see Figure 3aVII). On the other hand, the adjacent amphid wing (AWC) cilia have a fan-like morphology at the tip (Figure 3aVIII), and recognize volatile odorants [204]. Interestingly, in these sensory cilia, heterotrimeric kinesin II and homodimeric osmotic avoidance abnormal protein 3 (OSM-3/KIF 17), are required to transport the same IFT particles. However, in amphid channel cilia, while Kinesin II is required to transport them through the middle region of the axoneme (9+ few central singlets pattern), OSM-3 transports them along the distal region where only singlets, extensions of the A-tubules, are present [204]. On the other hand, in AWC, the kinesin II and OSM-3 kinesin motors are functionally redundant [204]. Mutations in OSM-3 cause a decrease in amphid channel cilia length, as a consequence of distal singlets’ loss. However, they have no apparent impact on the morphology of wing cilia [204]. This shows that in certain cilia the existence of a singlet/A-tubules’ subdomain (characterized by a variety of numbers and lengths), is critical for cilia function. Another example of the importance of this singlet subdomain is evident during the *Chlamydomonas* mating process where the structure of the flagellar tips changes. During mating, the tip accumulates dense material containing adhesive complexes (agglutinins) [125]. Subsequently, the A-microtubules elongate, growing into the distal region of the tip and increasing the tip segment length by 30%. These alterations seem to be required for sexual signaling [205]. In agreement, *Tetrahymena* FAP256/CEP104-KO cells, which show shorter A-tubules and reduction in the length of the distal segment, present decreased ability to conjugate (form pairs) [175]. This seems to be due to lower levels and decreased cortical presence of mating type proteins. In these examples it is unequivocal that the existence of a singlet region at the cilia tip is required for the reception of different types of signals, as well as their transduction. It is plausible regulating the dynamics of A-tubules in their singlet segment, will allow tips to adapt/respond better to environmental signals. In the cilia of *Tetrahymena* and *Chlamydomonas*, where a central pair is present along the whole cilium, the singlets’ segment will allow the construction of a less rigid and flexible region, counterbalancing the rigidity conferred by the central microtubules. This will be also true for the tips of cilia ending in a singlet region without any cap structure, as is the case of primary cilia. In this last case, by comparison, the tip will be much more flexible. Indeed, in many cilia with this type of tips, the singlets seem to get entangled, which probably allows interactions between them and the stabilization of the tip.

Additionally, the existence of a small region of singlets may favor the turn-over of distinct structural components of the axoneme, namely the axonemal B-tubules, by allowing them to assemble/repair at the end of the middle region. It is now known that in *Chlamydomonas* the anterograde IFT trains are transported primarily on the B-tubule, whereas retrograde IFT trains are transported on the A-tubule [206]. At the tip, as already mentioned, they get rearranged, switch motors and change tracks [204]. Therefore, the existence of a singlet region may assure that delivered IFT particles have reached their target and should start rearrangement, exchange motor and find the way back in A-tubules. Still, and reflecting the fascinating diversity of eukaryotic cilia, some axonemes of putative sensory ones present no signs of singlets, ending in blunt doublets. One such case is that of the cilia of *Leishmania* amastigotes inside of the parasitophorous vacuole [92], which may reflect the environment and the type of signals it helps transduce. In fact, signal per se is required for the maintenance of cilia tip structures of several cilia. For example, in the structure of *C. elegans* AWB olfactory neuron cilia, especially cilia membrane biogenesis, is affected by levels of sensory signaling. When signaling decreases, the membraneous structures found at cilia tips expand and the distribution patterns of some transmembrane signaling molecules is altered [111].

Finally, the complete picture of the role of kinesins in cilia distal domains will require understanding of the mechanisms involved in their regulation, for example by kinases that play critical ciliary roles [191,207,208,209].

##### Other Proteins Associated with Microtubules

One example of a microtubule-associated protein localizing at the ciliary distal domain is that of the *Trypanosoma* calpain-like protein (TbCALP1.3) which localizes at the flagellum tip [210]. Remarkably, in vertebrates, calpains are a ubiquitous family of calcium-dependent cysteine proteases that do not destroy, but rather modulate the functions of their substrates, and are involved in a wide range of cell functions (for review [211]). However, in protozoans, numerous genes encoding atypical calpains have been identified. Like TbCALP1.3, these proteins lack the characteristic calcium-binding penta-EF-hand motif, and most do not show enzymatic activities. Additionally, the protein contains N-terminal dual myristoylation/palmitoylation signals, protein modifications that facilitate membrane associations. In fact, TbCALP1.3, is subject to acylation with palmitic acid in vivo [210]. It was also shown that, although this modification is not sufficient for targeting TbCALP1.3 to the flagellum tip, the deletion of the potentially acylated residues impairs this localization. The role of these proteins in cilia is unknown. However, there are several evidences that calpain-like proteins behave as microtubule-interacting proteins, playing roles in microtubule cytoskeleton organization in mammals and Trypanosomes [212,213]. For example, mouse calpain-6 (Capn6), which lacks protease activity, is a microtubule-stabilizing protein, expressed in embryonic tissues, and that may be involved in the regulation of microtubule dynamics and cytoskeletal organization [212]. Depletion of Capn6 in NIH 3T3 human cells causes microtubule instability with a loss of acetylated-tubulin, and induced actin reorganization [212].

###### 2.4.3. Channel Proteins and Cilia Signaling Transduction

Other proteins that localize at the ciliary tip, and are related to sensory cilia functions are Polycystins. Mutations in the polycystin genes, PKD1 or PKD2, result in Autosomal Dominant Polycystic Kidney Disease (ADPKD) (for review [214]). Polycystin-2 (encoded by PKD2) is a member of the large 6-transmembrane spanning transient receptor potential (TRP) ion channel family, which modulates calcium release from the endoplasmic reticulum, and has been observed to form a complex with polycystin-1 (PKD1) [215]. It has been suggested that these complexes mediate calcium influx in response to fluid flow, and are localized in the endoplasmic reticulum membrane, the plasma membrane, exosomes and primary cilia (for review [214]). The polycystin-2 homolog of Drosophila (AMO, almost there) localizes to the tip of the sperm flagellum [216]. A mutation in this gene causes the absence of AMO from the tip, and nearly complete male sterility as the sperm is unable to fertilize the egg although able to swim. The AMO protein modulates flagellar beating and guides a backward trajectory into the sperm storage organs. Consequently, its absence impairs sperm storage. The authors postulated that its localization makes AMO privileged to receive cues (probably ligand molecules) upon transfer to the female reproductive tract that are critical for triggering PC2-mediated signaling [216].

Another protein channel localizing at cilia tips are the No mechanoreceptor potential C (TRPN/NOMPC) subunits, which are strong candidates to play roles as mechanotransducers in both vertebrates and invertebrates. In Drosophila, NOMPC is required to generate mechanoreceptor potentials and currents in tactile bristles [217]. NOMPC, together with a Transient receptor potential cation channel subfamily V (TRPV), is involved in signal transduction by the chordotonal neurons of the fly’s antennal ear, but both have distinct functions. Interestingly, NOMPC localizes in external and chordotonal sensory neurons at the tips of mechanosensory cilia. Additionally, in chordotonal sensory neurons, the TRPN and TRPV channels localize differentially into distal and proximal ciliary zones, respectively. Consistent with a sensory function, in *Xenopus*, a TRPN1 channel is also highly abundant in the terminal bulbs of the kinocilium in sensory hair cells. This TRPN1 localization is lost upon treatment with ethylene glycol-bis(β-aminoethyl ether)-*N*,*N*,*N*′,*N*′-tetraacetic acid (EGTA), concentrating then at the base of the cilium, which supports its involvement in signal transduction [218].

###### 2.4.4. Post-Translational Modifications at the Cilia Distal Domain

Proteins with methyl arginine residues are enriched at the tip and base of flagella, and their localization changes during flagellar assembly and disassembly [219]. In *Chlamydomonas*, 4 protein arginine methyltransferases (PRMTs) localize through the axoneme in a punctate pattern and show accumulation at the flagella base and enrichment at the flagellar tip [219]. The levels of PRMT1, but not those of PRMT3 (both enzymes add two methyl groups to one of the guanidino nitrogens of arginine), increase considerably at the tip during flagellar regrowth, which correlates with the dimethyl arginine modifications that take place there during this event. PRMTs are transported by IFT along the axoneme, and it was proposed that methylation of IFT proteins may decrease their affinity for tubulin, which will allow cargo unloading [219]. 

###### 2.4.5. Cap Structural Proteins

Until now, the only structural protein identified as a component of cap structures is Sentan. As already mentioned, human respiratory and oviductal cilia have specific caps, presenting a narrowed distal portion and a ciliary crown. In these cilia, Sentan localizes exclusively between the ciliary membrane and the peripheral singlet microtubules at the distal narrowed portion, suggesting that it is involved in bridging singlets to the ciliary membrane [220]. Sentan starts to localize at the tip when fetal trachea cilia reach 3 μm in length, which is concomitant with the establishment of a singlets’ (A-tubules) domain. Expression of Sentan in HeLa cells showed that this protein has affinity for membrane protrusions, and for binding to phosphatidylserine [220]. Interestingly, Sentan seems to be a specific component of tracheal and oviductal motile cilia caps, since it is not detectable in kidney and RPE 1 cells’ primary cilia, and it is not expressed in testis.

A proteomic analysis of a trypanosome mutant strain possessing a flagellum detached from the cell body, except for the basal body anchoring, revealed the presence of new flagella proteins, FLAM (Flagellum Members). These proteins showed to have specific localizations in the flagella, and FLAM 8 only accumulates at the tip of the peripheral microtubule doublets [221]. During flagellum elongation, FLAM 8 was only visible at the tip at advanced stages of flagellum assembly. However, the levels of FLAM8 in the new flagella only reach the levels similar to those found in the old flagella after cell division. FLAM8 localization is resistant to detergent treatments, which suggests that it must be part of a specific structure at the tip of the *Trypanosoma* flagella [221].

The specific localization of a few proteins in specific association only with singlets (A-tubule) raises the question of how they distinguish the microtubules in the singlet region from those present in doublet region (middle domain). Kubo et al. [220] suggested that this may due to differential distribution of post-translational modifications that are known to occur throughout the cilia compartment. For example, tubulin monomeric glycylation does not occur at the ciliary tip [222]. It is plausible that singlets harbor specific post-translational modifications that may be directly recognized by proteins such as Sentan, or indirectly, involving other specific microtubule-associated proteins.

**Figure 5:**
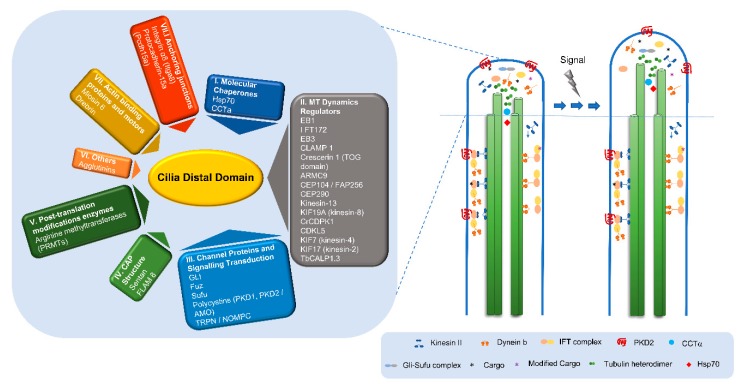
The dynamic distal domain and associated proteins. Important events in cilia function occur at their distal domain like cilia assembly/disassembly, intraflagellar transport (IFT) complexes’ remodeling, and signal reception/transduction. The IFT system forms cargo-carrying trains moved along the axoneme through the action of kinesin-2 (anterograde) and dynein-2 (retrograde) motors. This transport is essential for the assembly and maintenance of cilia and flagella. Tubulin subunits are transported throughout the cilium by IFT. At the tip, IFT trains split apart and mix with each other to assemble into new trains, which move back to the cell body with new cargos. Kinesin transports dynein to the tip as inactive cargo, disconnects from IFT trains at the tip and turns around by diffusion. Distinct functional arrays of ciliary proteins that concentrate at the tip are indicated (Molecular chaperones, Microtubule dynamic regulators, Channel proteins and signaling transduction, Cap structural proteins (when cap structures are present), Post-translational modifications enzymes, Actin binding proteins and motors, Anchoring junctions and others). A-tubule singlets are in general characteristic of the distal domain and present a dynamic behavior by polymerization and depolymerization, therefore changing the tip segment length in response to different signals.

In summary, the current knowledge on the molecular composition of the cilia tip (Figure 5) clearly shows that there is an accumulation of numerous proteins with microtubule-associated roles in this compartment. This reflects the dynamic nature of several processes that take place there, and which require a strict regulation of microtubule organization, post-translation modifications, association with multiple cellular machineries, and overall dynamics. Importantly, different environmental cues and stimuli impact on the intricate molecular pathways regulated at the ciliary tip, allowing for cilia-dependent signaling. The accumulation of different types of molecular chaperones in this domain indicates that they may assist in the turn-over, and/or remodeling of cilia components required for the maintenance of the organelle, and also as a response to signaling events. As expected, several proteins that localize at the cilia tip are components of signaling pathways, supporting this as a critical compartment for the concentration of signals and triggering of signaling cascades. Less studied are the enzymes responsible for post-translational modifications at ciliary tips. These modifications are exceptional candidates to create combinatory codes to be read by proteins residing or in transit at the tip, and that can play critical roles as transducers or effectors in signaling pathways. 

While a role for microtubule dynamics in the organization of cilia distal segments has been the focus of many studies, the role of the tip membrane domain has been neglected. However, in this region, not only A-microtubules are frequently linked to the membrane, but it is expected that receptors and transducers concentrate/organize at this membrane domain. Interestingly, during *Trypanosoma* division each cell assembles a new flagellum while maintaining the existing one. In this process, members of the kinesin family are also involved in the attachment of microtubules’ ends to flagella tip membranes [112]. In plants, kinesins, in coordination with kinases, originate signaling modules required for phragmoplast expansion and cell-plate growth [223,224]. Therefore, it is tempting to suggest that microtubules help organize membrane functional domains specialized in sensing and transducing at the tip or vice-versa. Similarly to what occurs with integrins’ activation [225], the activation of receptors at the tip may determine the targeting of microtubules to the membrane by concentrating proteins associated with microtubule-based functions in their vicinity. This microtubule-membrane interaction may be required for triggering signal pathways. Noteworthy, in zebrafish hair cells the complex Itga8 (integrin α8)–Pcdh15a (protocadherin-15a) accumulates at the kinocilium tip and its loss of function causes defects in cilia elongation and endocytosis impairment [226]. It seems that the Itga8–Pcdh15a complex acts through the activation of Ras homolog gene family, member A (RhoA), and possibly by the regulation of actin cytoskeleton dynamics. Recently, Nachury group [227] has shown that signaling G protein-coupled receptors exit the cilia by tip ectocytosis. The receptors concentrate into membranous buds at the cilia tips before release into extracellular vesicles (ectosomes). This process is regulated by actin and the actin regulators drebrin, myosin 6, Arp2/3 (Actin-Related Proteins) complex, and α-actinin 4 and is required for the correct regulation of Hh signaling [227]. In this scenario, it is possible that the Itga8–Pcdh15a complex may also play a role in this process through the activation of RhoA. These recent findings tell us that besides the microtubule cytoskeleton, actin also plays important roles at the tips of cilia that have only now started to be uncovered. 

## 3. Concluding Remarks

Although cilia have fascinated scientists since their discovery over a century ago, and the fact that the last 20 years have been characterized by the intensive study of these organelles, they are still revealing unsuspected structural and functional features. Structurally, the well-accepted canonical pattern of (9+2) and (9+0) for axonemal organization is full of exceptions, not only throughout of the eukaryotic phylogenetic tree, but also within the same organism or even a single cell. Moreover, this diversity extends to the base and distal ciliary domains.

We are far from understanding all the specific roles of the ciliary tip domain and their underlying mechanisms. Importantly, we do not know how specific functions are correlated with a specific cilia tip architectures and the regulation of cellular machineries like the microtubule and actin cytoskeletons. A critical step for the study of this, and other ciliary domains, is their molecular characterization in different cilia types. This is particularly relevant in the case of ciliary cap structures whose components are almost completely unknown, which has prevented their functional characterization. This will allow us to complement our view on how each cilia type is assembled and maintained, and establish a relationship for instance between the structural organization of ciliary tips and specific signaling events occurring there. One of the layers of complexity that likely plays a role in the establishment of distinct cilia tip architectures and functions has to do with the diversity of tubulins, conferred by the expression of different isotypes as well as by multiple post-translation modifications. All these factors contribute to determine the overall dynamic behavior of microtubules, their association with motor proteins, which could potentially impact on IFT, and likely also the cross-talk between microtubules and other cellular structures such as other cytoskeleton components and membranes. However, considering the importance of tubulin isotype diversity, the knowledge on its contribution to of cilia biology is lagging.

In addition, the recent evidences that ciliary integrins and cadherin-like molecules accumulate at the ciliary distal domain, and the fact that their action involves actin and actin regulators, will deserve more attention. These new results open a new window to the understanding on how receptors mediating cilia signal transduction operate. Indeed, the cross-talk between ciliary actin and microtubules to regulate these processes should also be under focus. Interestingly, in recent years the shedding of extracellular vesicles—including exosomes and microvesicles—by cells has emerged as a form of intercellular communication. Excitingly, recent data shows that cilia use these mechanisms at their tips in order to communicate and regulate signaling. A better understanding of these and other aspects of cilia biology relying on the distal compartment will come from further studies on different cilia types to elucidate their molecular composition, biogenesis, and functions. This will allow us to identify new ciliopathy genes, as well as clarify the heterogeneous phenotypes of ciliopathies.

## Figures and Tables

**Table 1:** Examples of variations to the (9+2) and (9+0) axoneme patterns.

Organism	Axoneme Pattern	Function	Reference
*Lecudina tuzetae* (*Miozoa*; parasitic protozoa)	(6+0)	Motile flagellum	[70,90]
*Diplauxis hatti* (*Miozoa*; parasitic protozoa)	(3+0)	Motile flagellum	[91]
*Leishmania mexicana* amastigotes (*Euglenozoa*; parasitic protozoa)	(8+1) or (7+2)	Immotile Flagellum	[92]
*Caenorhabditis* *Elegans* (*Nematoda*)	(9+0) changing transiently to 18–22 singlet microtubules at middle region, consequence of doublet splaying	Sensory cilia (Cephalic male (CEM) neurons)	[87]
Several species of *Arthropoda*	(12+0); (14+0); (16+0); (9+9+2)	Motile and non-motile spermatozoa	[89,93,94]
*Rattus* (*Chordata*)	(8+1)	Immotile cilia (Cerebral cortex)	[95]

**Table 2:** Examples of different distal domain structures.

Organism	Plugs	Central MT ^1^ Cap	Ciliary Crown	Other	References
*Chlamydomonas* (green alga) *Tetrahymena* (body cilia, ciliate protozoa) *Aequipecten* (bay scallop gill)	Plug structures are inserted into the tip of the A-tubules of the outer doublets and attached to the membrane by distal filaments	Linked to the membranes	No		[15,104,107,108]
*Tetrahymena* (oral apparatus cilia, ciliate protozoa) *Aequipecten* (certain cilia, bay scallop gill)	Plug structures are inserted into the tip of the A-tubules of the outer doublets and attached to the central microtubule cap by distal filaments	Linked to the membranes	No		[104]
*Crithidia, Herpetomonas,* *Trypanosoma and Leishmania* (Promastigote; parasitic protozoa)	No	No	No	Blunt end with two dense material regions, one associated with the MT central pair and the other with the doublets	[113]
*Leishmania* (Amastigote, parasitic protozoa)	No	No	No	No dense material or organized structure	[92]
*Beroe* (macrocilia, ctenophore)	No	No	No	Giant capping structure at the tip, formed by extensions of the A and central-pair MTs, bound together by amorphous material	[110]
*Paratomella* (haptocilia, flat worm)	No	No	Structure resemble crown	Asymmetrical cap structure. Some of the doublets end as a single A-microtubule, whereas for others, the A- and B-microtubules terminate in the cap. MT central pair and doublets 1, 2, 3, 8 and 9 terminate in a distal cap, whereas doublets 4 to 7 end at a dense material of a proximal secondary cap	[103,119]
*Caenorhabditis* (sensory amphid channel, worm)	No	No	No	Tip presents only MT singlets. No cap or dense material	[111]
*Caenorhabditis* (sensory amphid wing cilia C, worm)	No	No	No	The middle region of the axoneme both contain doublets and singlets that “splay apart” laterally and end together. The tip presents a membranous fan-like structure. No cap or dense material	[111]
*Lima* (bivalve)	No	No	No	Dense material at the distal ends of the axonemal MTs	[122]
*Periplaneta* (antenna pedicel, cockroach)	No	No	No	Dilated tip, doublets become singlets and terminate in an electron-dense spheroid	[123]
*Bombina* (frog)	Plugs are inserted into the lumen of the A-tubules, attaching them to the caps	No	No	Asymmetrical cap structure. One large cap, linked to the membrane and to doublet MTs number 4 to 7, and a smaller cap linked to the doublets number 1, 2, 3, 8 and 9, as well as to the two central MTs	[121]
Primary cilia (vertebrates)	No	No	No	In distal segment doublets are converted into singlets through the loss of the B-tubule. No cap or dense material	[124]

^1^ Microtubules.
